# Supplementary material for: The epidemiology of behavioral risk factors for noncommunicable disease and hypertension: A cross-sectional study from Eastern Uganda
Source: PLOS Glob Public Health. 2024 Jun 17;4(6):e0002998. doi: 10.1371/journal.pgph.0002998 (PMC11182527; doi:10.1371/journal.pgph.0002998)
Supplement: S1 Table — (DOCX) [file pgph.0002998.s002.docx]

**S1 Table.** Demographic characteristics of study participants; Iganga-Mayuge, Uganda (Nov 2017-June 18)

| **Indicator** | **Females**  **(n=1716)** | **Males**  **(n=1504)** | **Total**  **(n=3220)** |
| --- | --- | --- | --- |
| **Age category (years)** |  |  |  |
| 18-29 | 374 (21.8) | 365 (24.3) | 739 (23.0) |
| 30-44 | 392 (22.8) | 375 (24.9) | 767 (23.8) |
| 45-59 | 465 (27.1) | 361 (24.0) | 826 (25.7) |
| >60 | 485 (28.3) | 403 (26.8) | 888 (27.6) |
| **Education** |  |  |  |
| None | 448 (26.1) | 142 (9.4) | 590 (18.3) |
| Primary | 791 (46.1) | 735 (48.9) | 1526 (47.4) |
| Secondary | 384 (22.4) | 493 (32.8) | 877 (27.2) |
| More than secondary | 93 (5.4) | 134 (8.9) | 227 (7.1) |
| **Marital Status** |  |  |  |
| Never married | 161 (9.4) | 280 (18.6) | 441 (13.7) |
| Married | 991 (57.8) | 1109 (73.7) | 2100 (65.2) |
| Divorced/separated | 162 (9.4) | 81 (5.4) | 243 (7.6) |
| Widowed | 402 (23.4) | 34 (2.3) | 436 (13.6) |
| **Location** |  |  |  |
| Rural | 1125 (65.6) | 977 (65.0) | 2102 (65.3) |
| Peri-urban | 590 (34.4) | 527 (35.0) | 1117 (34.7) |

Data are n (%).
