# Supplementary material for: The epidemiology of behavioral risk factors for noncommunicable disease and hypertension: A cross-sectional study from Eastern Uganda
Source: PLOS Glob Public Health. 2024 Jun 17;4(6):e0002998. doi: 10.1371/journal.pgph.0002998 (PMC11182527; doi:10.1371/journal.pgph.0002998)
Supplement: S2 Table — (DOCX) [file pgph.0002998.s003.docx]

**S2 Table.** Crude and adjusted risk ratios for hypertension in females

| **Variable** |  | **n** | **# hypertensive (%)** | **Crude RR** | **Adjusted RR** |
| --- | --- | --- | --- | --- | --- |
| **Age (years)** | 18-29 | 329 | 15 (4.6) | Ref. | Ref. |
|  | 30-44 | 353 | 63 (17.9) | **4.30 (2.45-7.5)** | **3.80 (2.18-6.61)** |
|  | 45-59 | 464 | 159 (34.3) | **8.39 (4.95-14.2)** | **7.62 (4.48-13.0)** |
|  | 60+ | 480 | 265 (55.2) | **13.8 (8.23-23.11)** | **14.0 (8.39-23.4)** |
| **Education** | None | 437 | 193 (44.2) | Ref. | Ref. |
|  | Primary | 746 | 227 (30.4) | **0.57 (0.46-0.70)** | 1.00 (0.81-1.24) |
|  | Secondary | 357 | 65 (18.2) | **0.31 (0.23-0.43)** | 0.86 (0.63-1.19) |
|  | > Secondary | 86 | 17 (19.8) | **0.25 (0.14-0.44)** | 0.60 (0.34-1.05) |
| **Location** | Rural | 1061 | 337 (31.8) | Ref. | Ref. |
|  | Peri-urban | 565 | 165 (29.2) | 1.02 (0.81-1.27) | **1.33 (1.08-1.64)** |
| **Current tobacco use** | No | 1603 | 492 (30.7) | Ref. | Ref. |
|  | Yes | 23 | 10 (43.5) | 1.55 (0.80-3.01) | 0.82 (0.46-1.46) |
| **Current drinker (30 days)** | No | 1534 | 473 (30.8) | Ref. | Ref. |
|  | Yes | 92 | 29 (31.5) | 1.39 (0.92-2.12) | 0.67 (0.32-1.42) |
| **Heavy episodic drinking** | No | 1553 | 479 (30.8) | Ref. | Ref. |
|  | Yes | 73 | 23 (31.5) | 1.42 (0.90-2.24) | 1.48 (0.63-3.46) |
| **Low fruit & vegetable** | No | 35 | 8 (22.9) | Ref. | Ref. |
| **consumption**† | Yes | 1591 | 494 (31.1) | 1.81 (0.83-3.95) | **2.14 (1.10-4.16)** |
| **Add salt while eating** | Never/rarely | 1121 | 365 (32.6) | Ref. | Ref. |
|  | Sometimes | 454 | 117 (25.8) | **0.76 (0.58-0.98)** | 1.00 (0.78-1.28) |
|  | Often/always | 51 | 20 (39.2) | 0.91 (0.51-1.62) | 1.33 (0.80-2.23) |
| **Add salt while cooking** | Never/rarely | 471 | 169 (35.9) | Ref. | Ref. |
|  | Sometimes | 576 | 183 (31.8) | 0.81 (0.63-1.05) | 1.00 (0.79-1.26) |
|  | Often/always | 577 | 148 (25.7) | **0.73 (0.56-0.94)** | 1.29 (0.99-1.66) |
| **Eat processed foods** | Never/rarely | 852 | 289 (33.9) | Ref. | Ref. |
| **high in salt** | Sometimes | 679 | 192 (28.3) | **0.74 (0.59-0.92)** | 0.90 (0.73-1.11) |
|  | Often/always | 95 | 21 (22.1) | 0.68 (0.39-1.19) | 0.89 (0.52-1.53) |
| **Insufficient physical** | No | 1401 | 400 (28.6) | Ref. | Ref. |
| **activity**‡ | Yes | 225 | 102 (45.3) | **1.41 (1.08-1.85)** | 0.99 (0.79-1.24) |
| **BMI (kg/m^2^)** | <25.0 | 846 | 220 (26.0) | Ref. | Ref. |
|  | 25.0-29.9 | 474 | 142 (30.0) | **1.36 (1.05-1.77)** | **1.30 (1.02-1.65)** |
|  | >30 | 278 | 125 (45.0) | **2.52 (1.96-3.25)** | **1.87 (1.46-2.40)** |

Pregnant women are excluded from analysis. Bolded risk ratios and 95% confidence intervals indicate statistical significance at p<0.05. †Defined as those who ate less than 5 servings of fruit and/or vegetables on average per day**.** ‡Defined as not achieving 150 minutes of moderate-intensity physical activity OR 75 minutes of vigorous-intensity physical activity OR an equivalent combination of moderate- and vigorous-intensity physical activity achieving at least 600 MET-minute. Abbreviations: BMI, body mass index; kg, kilogram; m, meter.
