# Supplementary material for: The epidemiology of behavioral risk factors for noncommunicable disease and hypertension: A cross-sectional study from Eastern Uganda
Source: PLOS Glob Public Health. 2024 Jun 17;4(6):e0002998. doi: 10.1371/journal.pgph.0002998 (PMC11182527; doi:10.1371/journal.pgph.0002998)
Supplement: S3 Table — (DOCX) [file pgph.0002998.s004.docx]

**S3 Table** Crude and adjusted risk ratios for hypertension in males

| **Variable** |  | **n** | **# hypertensive (%)** | **Crude RR** | **Adjusted RR** |
| --- | --- | --- | --- | --- | --- |
| **Age (years)** | 18-29 | 365 | 23 (6.3) | Ref. | Ref. |
|  | 30-44 | 374 | 73 (19.5) | **3.01 (1.92-4.74)** | **2.69 (1.70-4.28)** |
|  | 45-59 | 359 | 102 (28.4) | **4.42 (2.86-6.84)** | **3.97 (2.52-6.27)** |
|  | 60+ | 403 | 201 (49.9) | **7.83 (5.19-11.8)** | **6.63 (4.22-10.4)** |
| **Education** | None | 140 | 52 (37.1) | Ref. | Ref. |
|  | Primary | 734 | 211 (28.8) | **0.65 (0.46-0.91)** | 1.11 (0.78-1.58) |
|  | Secondary | 493 | 94 (19.1) | **0.41 (0.28-0.60)** | 1.02 (0.68-1.52) |
|  | > Secondary | 134 | 42 (31.3) | 0.81 (0.52-1.27) | 1.14 (0.69-1.88) |
| **Location** | Rural | 974 | 278 (28.5) | Ref. | Ref. |
|  | Peri-urban | 527 | 121 (23.0) | 0.86 (0.67-1.11) | 0.98 (0.76-1.28) |
| **Current tobacco use** | No | 1335 | 341 (25.5) | Ref. | Ref. |
|  | Not daily | 166 | 58 (34.9) | **2.13 (1.58-2.86)** | 1.20 (0.87-1.65) |
| **Current drinker (30 days)** | No | 1300 | 312 (24.0) | Ref. | Ref. |
|  | Yes | 201 | 87 (43.3) | **2.54 (1.96-3.29)** | **1.92 (1.13-3.24)** |
| **Heavy episodic drinking** | No | 1339 | 327 (24.4) | Ref. | Ref. |
|  | Yes | 162 | 72 (44.4) | **2.36 (1.78-3.13)** | 1.02 (0.59-1.78) |
| **Low fruit & vegetable** | No | 20 | 8 (40.0) | Ref. | Ref. |
| **consumption**† | Yes | 1481 | 391 (26.4) | 0.62 (0.28-1.40) | 0.88 (0.41-1.90) |
| **Add salt while eating** | Never/rarely | 921 | 254 (27.6) | Ref. | Ref. |
|  | Sometimes | 507 | 122 (24.1) | 0.88 (0.68-1.14) | 1.12 (0.86-1.45) |
|  | Often/always | 73 | 23 (31.5) | **1.64 (1.03-2.60)** | **1.64 (1.01-2.70)** |
| **Add salt while cooking** | Never/rarely | 304 | 98 (32.2) | Ref. | Ref. |
|  | Sometimes | 576 | 144 (25.0) | **0.61 (0.44-0.82)** | **0.68 (0.50-0.93)** |
|  | Often/always | 613 | 157 (25.6) | **0.73 (0.54-0.98)** | 0.88 (0.65-1.20) |
| **Eat processed foods** | Never/rarely | 638 | 195 (30.6) | Ref. | Ref. |
| **high in salt** | Sometimes | 713 | 170 (23.8) | 0.85 (0.66-1.08) | 1.11 (0.87-1.42) |
|  | Often/always | 150 | 34 (22.7) | 0.79 (0.51-1.21) | 1.13 (0.72-1.78) |
| **Insufficient physical** | No | 1361 | 332 (24.4) | Ref. | Ref. |
| **activity**‡ | Yes | 140 | 67 (47.9) | **2.52 (1.87-3.40)** | **1.60 (1.15-2.22)** |
| **BMI (kg/m^2^)** | <25.0 | 1033 | 228 (22.1) | Ref. | Ref. |
|  | 25.0-29.9 | 367 | 119 (32.4) | **1.48 (1.14-1.93)** | **1.30 (1.01-1.66)** |
|  | >30 | 84 | 45 (53.6) | **3.62 (2.66-4.94)** | **2.23 (1.57-3.18)** |

Bolded risk ratios and 95% confidence intervals indicate statistical significance at p<0.05. †Defined as those who ate less than 5 servings of fruit and/or vegetables on average per day**.** ‡Defined as not achieving 150 minutes of moderate-intensity physical activity OR 75 minutes of vigorous-intensity physical activity OR an equivalent combination of moderate- and vigorous-intensity physical activity achieving at least 600 MET-minute. Abbreviations: BMI, body mass index; kg, kilogram; m, meter
