# Supplementary material for: The epidemiology of behavioral risk factors for noncommunicable disease and hypertension: A cross-sectional study from Eastern Uganda
Source: PLOS Glob Public Health. 2024 Jun 17;4(6):e0002998. doi: 10.1371/journal.pgph.0002998 (PMC11182527; doi:10.1371/journal.pgph.0002998)
Supplement: S4 Table — (DOCX) [file pgph.0002998.s005.docx]

**S4** Table Comparison of 2014 national survey estimates with IM-HDSS estimates for NCD risk factors in males by age

|  | **18-29 years** | | **30-49 years** | | **50-69 years** | |
| --- | --- | --- | --- | --- | --- | --- |
| **Indicator** | **2014 STEPS** | **IM-HDSS** | **2014 STEPS** | **IM-HDSS** | **2014 STEPS** | **IM-HDSS** |
| **Tobacco use** |  |  |  |  |  |  |
| Current smoker | 7.9 (5.4-10.5) | 3.0 (1.5-5.3) | 21.4 (17.4-25.5) | 11.8 (9.1-14.8) | 29.4 (21.2-37.6) | 15.6 (12.2-19.6) |
| Current smokeless | 2.9 (1.1-4.7) | 1.6 (0.6-3.5) | 4.2 (2.3-6.0) | 1.5 (0.7-3.0) | 9.7 (4.9-14.5) | 0.0 (0.0-0.9) |
| **Alcohol intake** |  |  |  |  |  |  |
| Current drinker | 29.3 (24.6-33.9) | 7.7 (5.2-10.9) | 46.3 (41.2-51.4) | 13.9 (11.0-17.1) | 53.8 (45.5-62.0) | 16.9 (13.3-20.9) |
| Heavy episodic drinking* | 17.6 (14.0-21.2) | 7.1 (4.7-10.3) | 32.4 (27.6-37.2) | 11.0 (8.4-14.0) | 34.3 (25.4-43.1) | 12.8 (9.7-16.5) |
| **Diet** |  |  |  |  |  |  |
| Low fruit & vegetable consumption† | 88.6 (85.2-91.9) | 98.4 (96.5-99.4) | 89.8 (86.7-92.9) | 97.9 (96.4-98.9) | 84.6 (79.2-90.0) | 97.4 (95.7-98.6) |
| Always/often add salt while eating | 24.6 (19.9-29.3) | 5.5 (3.4-8.3) | 24.9 (20.7-29.1) | 4.8 (3.1-7.0) | 21.6 (14.9-28.3) | 4.5 (2.7-7.1) |
| Always/often add salt while cooking | 43.8 (38.4-49.1) | 55.9 (50.6-61.1) | 44.4 (39.3-49.5) | 43.9 (39.6-48.3) | 31.8 (23.5-40.2) | 33.5 (28.9-38.4) |
| Always/often eat processed foods high in salt | 6.8 (4.8-8.8) | 17.5 (13.8-21.8) | 5.0 (3.0-6.9) | 10.4 (7.9-13.3) | 2.3 (0.0-4.7) | 5.5 (3.5-8.3) |
| **Physical activity** |  |  |  |  |  |  |
| Insufficient physical activity‡ | 2.1 (0.7-3.5) | 3.3 (1.7-5.8) | 3.1 (1.7-4.6) | 6.4 (4.4-8.8) | 9.3 (1.1-17.5) | 8.3 (5.8-11.5) |
| **Body Mass Index (BMI; kg/m^2^)** § |  |  |  |  |  |  |
| Underweight (BMI< 18.5) | 5.6 (3.5-7.7) | 1.6 (0.6-3.6) | 10.7 (7.5-13.9) | 2.1 (1.1-3.8) | 20.9 (14.0-27.7) | 4.4 (2.6-6.9) |
| Normal (>18.5 BMI < 25) | 86.4 (83.1-89.7) | 79.1 (74.6-83.2) | 74.3 (74.2-78.5) | 63.2 (58.9-67.4) | 67.6 (59.4-75.9) | 58.0 (52.9-63.0) |
| Overweight (>25 BMI < 30) | 7.1 (4.3-9.9) | 17.9 (14.1-22.2) | 12.1 (9.2-15.0) | 28.3 (24.4-32.4) | 9.9 (4.9-14.9) | 27.8 (23.4-32.6) |
| Obese (BMI >30) | 0.9 (0.1-1.7) | 1.4 (0.4-3.2) | 2.8 (1.1-4.6) | 6.4 (4.4-8.9) | 1.6 (0.0-3.6) | 9.8 (7.0-13.2) |
| **Blood pressure field reading**§** |  |  |  |  |  |  |
| Hypertension | 18.2 (14.2-22.1) | 6.3 (4.0-9.3) | 26.1 (21.7-30.5) | 21.7 (18.2-25.5) | 41.7 (33.6-49.8) | 34.5 (29.8-39.4) |

Data are % (95%CI) and are from Uganda’s 2014 National STEPS survey and the present study (IM-HDSS). * Percent of population who had six or more alcoholic drinks in one sitting in the past month. †Defined as those who ate less than 5 servings of fruit and/or vegetables on average per day**.** ‡Defined as not achieving 150 minutes of moderate-intensity physical activity OR 75 minutes of vigorous-intensity physical activity OR an equivalent combination of moderate- and vigorous-intensity physical activity achieving at least 600 MET-minutes. § Excludes pregnant women. **Either SBP between 120 and 139 mmHg or DBP between 80 and 89 mmHg. Abbreviations: IM-HDSS, Iganga-Mayuge Health and Demographic Surveillance Site; kg, kilogram; m, meter.
