# Supplementary material for: The epidemiology of behavioral risk factors for noncommunicable disease and hypertension: A cross-sectional study from Eastern Uganda
Source: PLOS Glob Public Health. 2024 Jun 17;4(6):e0002998. doi: 10.1371/journal.pgph.0002998 (PMC11182527; doi:10.1371/journal.pgph.0002998)
Supplement: S5 Table — (DOCX) [file pgph.0002998.s006.docx]

**S5 Table** Comparison of 2014 national survey estimates with IM-HDSS estimates for NCD risk factors in females by age

|  | **18-29 years** | | **30-49 years** | | **50-69 years** | |
| --- | --- | --- | --- | --- | --- | --- |
| **Indicator** | **2014 STEPS** | **IM-HDSS** | **2014 STEPS** | **IM-HDSS** | **2014 STEPS** | **IM-HDSS** |
| **Tobacco use** |  |  |  |  |  |  |
| Current smoker | 0.2 (0.0-0.5) | 0.0 (0.0-0.9) | 2.3 (1.3-3.3) | 1.0 (0.4-2.2) | 12.3 (6.2-18.4) | 1.4 (0.5-2.8) |
| Current smokeless | 1.0 (0.1-1.8) | 0.2 (0.0-0.9) | 2.3 (1.1-3.4) | 0.4 (0.05-1.4) | 10.3 (4.3-16.4) | 1.2 (0.3-3.5) |
| **Alcohol intake** |  |  |  |  |  |  |
| Current drinker | 13.2 (9.6-16.7) | 1.9 (0.8-3.8) | 21.1 (17.6-24.6) | 6.2 (4.4-8.4) | 24.3 (19.2-29.5) | 8.0 (5.8-10.7) |
| Heavy episodic drinking* | 4.4 (2.7-6.1) | 1.9 (0.8-3.8) | 10.3 (7.6-12.9) | 5.7 (3.9-7.9) | 12.9 (8.7-17.0) | 6.0 (4.1-8.5) |
| **Diet** |  |  |  |  |  |  |
| Low fruit & vegetable consumption† | 88.2 (85.4-91.0) | 99.2 (97.6-99.8) | 86.2 (83.2-89.1) | 98.5 (97.0-99.3) | 87.5 (82.9-92.0) | 98.2 (96.4-99.3) |
| Always/often add salt while eating | 22.9 (18.9-26.8) | 5.3 (3.3-8.1) | 17.5 (14.6-20.5) | 2.7 (1.6-4.4) | 17.1 (11.1-23.1) | 2.5 (1.4-4.3) |
| Always/often add salt while cooking | 44.1 (39.5-48.7) | 56.7 (51.5-61.8) | 37.9 (33.5-42.2) | 37.6 (33.6-41.6) | 31.5 (25.7-37.3) | 28.8 (25.0-33.0) |
| Always/often eat processed foods high in salt | 5.2 (3.5-6.9) | 9.1 (6.4-12.5) | 4.4 (2.7-6.0) | 6.5 (4.7-8.8) | 1.2 (0.0-2.7) | 3.9 (2.4-6.0) |
| **Physical activity** |  |  |  |  |  |  |
| Insufficient physical activity‡ | 5.8 (4.0-7.7) | 9.4 (6.6-12.8) | 3.3 (1.9-4.6) | 6.7 (4.8-9.0) | 6.3 (3.6-9.0) | 9.2 (6.8-12.0) |
| **Body Mass Index (BMI; kg/m^2^)** § |  |  |  |  |  |  |
| Underweight (BMI< 18.5) | 4.8 (2.8-6.8) | 0.9 (0.2-2.6) | 6.8 (4.7-8.9) | 2.0 (1.0-3.6) | 12.1 (8.0-16.3) | 3.6 (2.1-5.5) |
| Normal (>18.5 BMI < 25) | 74.8 (71.1-78.5) | 62.5 (57.0-67.8) | 60.8 (56.9-64.7) | 42.7 (38.5-47.0) | 55.9 (49.2-62.7) | 46.4 (41.9-50.8) |
| Overweight (>25 BMI < 30) | 15.9 (12.6-19.1) | 28.7 (23.8-33.9) | 22.2 (19.0-25.4) | 31.9 (28.0-36.0) | 22.7 (16.4-29.0) | 30.4 (26.4-34.6) |
| Obese (BMI >30) | 4.5 (3.0-6.1) | 7.9 (5.2-11.4) | 10.2 (7.2-13.2) | 23.4 (19.9-27.2) | 9.2 (5.6-12.8) | 19.7 (16.3-16.2) |
| **Blood pressure field reading**§** |  |  |  |  |  |  |
| Hypertension | 11.9 (9.0-14.8) | 4.6 (2.6-7.4) | 22.9 (21.7-26.1) | 21.4 (18.0-25.1) | 46.2 (39.7-52.8) | 41.4 (37.1-45.8) |

Data are % (95%CI) and are from Uganda’s 2014 National STEPS survey and the present study (IM-HDSS). * Percent of population who had six or more alcoholic drinks in one sitting in the past month. †Defined as those who ate less than 5 servings of fruit and/or vegetables on average per day**.** ‡Defined as not achieving 150 minutes of moderate-intensity physical activity OR 75 minutes of vigorous-intensity physical activity OR an equivalent combination of moderate- and vigorous-intensity physical activity achieving at least 600 MET-minutes. § Excludes pregnant women. **Either SBP between 120 and 139 mmHg or DBP between 80 and 89 mmHg. Abbreviations: IM-HDSS, Iganga-Mayuge Health and Demographic Surveillance Site; kg, kilogram; m, meter.
