# Supplementary material for: The epidemiology of behavioral risk factors for noncommunicable disease and hypertension: A cross-sectional study from Eastern Uganda
Source: PLOS Glob Public Health. 2024 Jun 17;4(6):e0002998. doi: 10.1371/journal.pgph.0002998 (PMC11182527; doi:10.1371/journal.pgph.0002998)
Supplement: Attachment — Submitted filename: PGPH-D-23-02022_reviewer.pdf [file pgph.0002998.s010.pdf]

PLOS Global Public Health

The Epidemiology of Noncommunicable Disease Behavioral Risk Factors and Hypertension: A Cross-sectional Study from Eastern Uganda

--Manuscript Draft--

**Manuscript Number:**	PGPH-D-23-02022
**Article Type:**	Research Article
**Full Title:**	The Epidemiology of Noncommunicable Disease Behavioral Risk Factors and Hypertension: A Cross-sectional Study from Eastern Uganda
**Short Title:**	NCD Risk Factors in Eastern Uganda
**Corresponding Author:**	Gulam Muhammed Al Kibria<br>Johns Hopkins University Bloomberg School of Public Health<br>Baltimore, UNITED STATES
**Order of Authors:**	Dustin G Gibson, MS, PhD
	Ankita Meghani, PhD
	Charles Ssemagabo, PhD
	Adaeze Wosu, PhD
	Gulam Muhammed Al Kibria
	Tryphena Nareeba, MSc
	Collins Gyehazo, BSc
	Edward Galiwango, BSc
	George W Pariyo, PhD
	Judith Kaija Nanyonga
	Dan Kajungu, PhD
	Elizeus Rutebemberwa, PhD
	Adnan Ali Hyder, MD, MPH, PhD
**Keywords:**	Non communicable disease, hypertension, behavioral risk factors cross sectional study, Uganda
**Abstract:**	In light of the suboptimal noncommunicable disease (NCD) risk factor surveillance efforts, the study’s main objectives were to: (i) characterize the epidemiological profile of NCD risk factors; (ii) estimate the prevalence of hypertension; and (iii) identify factors associated with hypertension in a peri-urban and rural Ugandan population. A population-based cross-sectional survey of adults was conducted at the Iganga-Mayuge Health and Demographic Surveillance System site in eastern Uganda. After describing sociodemographic characteristics, the prevalence of NCD risk factors and hypertension was reported. Prevalence ratios for NCD risk factors were calculated using weighted Poisson regression to identify factors associated with hypertension.<br><br>Among 3220 surveyed respondents (mean age: 35.3 years (standard error: 0.1), 49.4% males), 4.4% (95% CI: 3.7-5.3) were current tobacco users, 7.7% (95% CI: 6.7-8.8) were current drinkers, 98.5% (95% CI: 97.9-98.9) had low fruit and vegetable consumption, 26.9% (95% CI: 25.0-28.8) were overweight, and 9.3% (95% CI: 8.2-10.5) were obese. There was a high prevalence of hypertension and prehypertension, at 17.1% (95% CI: 15.9-18.4) and 48.8% (95% CI: 46.8-51.0), respectively. Among hypertensive people, most had uncontrolled hypertension, at 97.4% (95% CI: 95.8-98.4). When we examined associated factors, older age (adjusted prevalence ratio (APR): 3.1, 95% CI: 2.2-4.4, APR: 5.2, 95% CI: 3.7-7.3, APR: 8.9, 95% CI: 6.4-12.5 among 30-44, 45-59, and 60+-year-old people than 18-29-year-olds), alcohol drinking (APR: 1.6, 95% CI: 1.3-2.0, ref: no), always adding salt during eating (APR: 1.6, 95% CI: 1.1-2.2, ref: no), poor physical activity (APR: 1.3, 95% CI: 1.1-1.6, ref: no), overweight (APR: 1.3, 95% CI: 1.1-1.5, ref: normal weight), and obesity (APR: 2.0,

	95% CI: 1.6-2.4, ref: normal weight) had higher prevalence of hypertension than their counterparts.<br><br>The high prevalence of NCD risk factors highlights the immediate need to implement and scale-up population-level strategies to increase awareness about leading NCD risk factors in Uganda. These strategies should be accompanied by concomitant investment in building health systems capacity to manage and control NCDs.
Additional Information:	
Question	Response
**Financial Disclosure**<br><br>Enter a financial disclosure statement that describes the sources of funding for the work included in this submission and the role the funder(s) played. This includes grants and any commercial funding of the work or authors.<br><br>This statement will be typeset if the manuscript is accepted for publication.<br><br>*Please review the submission guidelines and the instructions link below for detailed requirements and guidance.*	Research reported in this publication was supported by the Fogarty International Center of the National Institutes of Health under Award Number (DUNS Number001910777, Project # 1R21TW010415-01). The content is solely the responsibility of the authors and does not necessarily represent the official views of the Fogarty International Center nor the National Institutes of Health.
**Competing Interests**<br><br>On behalf of all authors, disclose any competing interests that could be perceived to bias this work.<br><br>This statement will be typeset if the manuscript is accepted for publication.<br><br>*Please review the instructions link below and PLOS Global Public Health's competing interests policy to determine what information must be disclosed at submission.*	The authors declare that they have no competing interests
**Data Availability**<br><br>Before publication, Authors are required to make fully available and without	Data will be available upon acceptance at https://www.openicpsr.org/openicpsr/workspace?path=openICPSR

restriction all data underlying their findings. Please see our PLOS Data Policy page for detailed information on this policy.<br><br>A **Data Availability Statement**, detailing where the data can be accessed, is required at first submission. Insert your Data Availability Statement in the box below.<br><br>Please see the data reporting section of our submission guidelines for instructions on what you need to include in your Data Availability Statement.<br><br>This statement will be typeset if the manuscript is accepted for publication.<br><br>*PLOS allows rare exemptions to address legal and ethical concerns. If you have legal or ethical restrictions, please use the box below to detail these in full sentences for the Journal team to consider.*	

Manuscript

Click here to access/download;Manuscript;MainText_Hypertension_NCD_Uga

The Epidemiology of Noncommunicable Disease Behavioral Risk Factors and Hypertension: A Cross-sectional Study from Eastern Uganda

Short title: NCD Risk Factors in Eastern Uganda

Dustin G Gibson, PhD; Ankita Meghani, PhD, MSPH[1]; Charles Ssemagabo, PhD; Adaeze Wosu, PhD, MPH; Gulam Muhammmed Al Kibria, PhD, MSPH, MBBS[*]; Tryphena Nareeba, MSc; Collins Gyezaho, BSc; Edward Galiwango, BSc; Judith Kaija Nanyonga; George W Pariyo, MBChB, PhD; Dan Kajungu, PhD ; Elizeus Rutebemberwa, PhD; Adnan Ali Hyder, MD, MPH, PhD

***Email:** gkibria1@jhu.edu

Abstract

In light of the suboptimal noncommunicable disease (NCD) risk factor surveillance efforts, the study's main objectives were to: (i) characterize the epidemiological profile of NCD risk factors; (ii) estimate the prevalence of hypertension; and (iii) identify factors associated with hypertension in a peri-urban and rural Ugandan population.

A population-based cross-sectional survey of adults was conducted at the Iganga-Mayuge Health and Demographic Surveillance System site in eastern Uganda. After describing sociodemographic characteristics, the prevalence of NCD risk factors and hypertension was reported. Prevalence ratios for NCD risk factors were calculated using weighted Poisson regression to identify factors associated with hypertension.

Among 3220 surveyed respondents (mean age: 35.3 years (standard error: 0.1), 49.4% males), 4.4% (95% CI: 3.7-5.3) were current tobacco users, 7.7% (95% CI: 6.7-8.8) were current drinkers, 98.5% (95% CI: 97.9-98.9) had low fruit and vegetable consumption, 26.9% (95% CI: 25.0-28.8) were overweight, and 9.3% (95% CI: 8.2-10.5) were obese. There was a high prevalence of hypertension and prehypertension, at 17.1% (95% CI: 15.9-18.4) and 48.8% (95% CI: 46.8-51.0), respectively. Among hypertensive people, most had uncontrolled hypertension, at 97.4% (95% CI: 95.8-98.4). When we examined associated factors, older age (adjusted prevalence ratio (APR): 3.1, 95% CI: 2.2-4.4, APR: 5.2, 95% CI: 3.7-7.3, APR: 8.9, 95% CI: 6.4-12.5 among 30-44, 45-59, and 60+-year-old people than 18-29-year-olds), alcohol drinking (APR: 1.6, 95% CI: 1.3-2.0, ref: no), always adding salt during eating (APR: 1.6, 95% CI: 1.1-2.2, ref: no), poor physical activity (APR: 1.3, 95% CI: 1.1-1.6, ref: no), overweight (APR: 1.3, 95% CI: 1.1-1.5, ref: normal weight), and obesity (APR: 2.0, 95% CI: 1.6-2.4, ref: normal weight) had higher prevalence of hypertension than their counterparts.

The high prevalence of NCD risk factors highlights the immediate need to implement and scale-up population-level strategies to increase awareness about leading NCD risk factors in Uganda. These strategies should be accompanied by concomitant investment in building health systems capacity to manage and control NCDs.

Key words: Non communicable disease, hypertension, behavioral risk factors cross sectional study, Uganda

Declarations of Interest: The authors have no conflicts of interest to declare.

Main Text

Introduction

Each year, over 41 million people globally die from noncommunicable diseases (NCDs), which equates to 73% of the global mortality burden and a 20% increase in the past 10 years [1]. Approximately two-thirds of these NCD deaths are attributed to four modifiable behavioral risk factors: physical inactivity, dietary risk, alcohol consumption, and tobacco use [1–4]. These risk factors, in addition to excessive salt intake, can contribute to raised blood pressure (BP) or hypertension, which is a major cause of all-cause deaths, estimated at 10.4 million in 2017 [3,4].

Uganda, like many low- and middle-income countries (LMICs), is undergoing an epidemiologic transition, shifting from a primary burden of infectious diseases towards preventable death and disability from NCDs [5–7]. NCDs are four of the ten most common causes of adult death, and among them, cardiovascular disease is the top cause, with age-standardized death rates of 338 per 100,000 people [8]. Raised BP, which is a leading cardiovascular risk worldwide, has also been a cause of growing public health concern in Uganda [9].

To quantify the magnitude and scope of NCD burden in Uganda, the national government, with the support of the World Health Organization (WHO) implemented its first nation-wide survey in 2014 to establish baseline prevalence estimates for NCD risk factors in the country as well as to examine the behaviors, knowledge, and practices of Ugandan communities regarding NCDs [9,10]. The results were striking: nearly one in ten Ugandans had more than three risk factors for NCDs, which included inadequate diet, low physical activity, being overweight or obese, or having raised BP. The survey results revealed a high burden of hypertension (26.4%), and further analyses showed a higher prevalence observed in urban areas (28.9%) compared to rural areas (25.8%), and a higher prevalence among men (28.3%) compared to women (25.2%). However, among those who had hypertension, roughly 8% were aware of their own status, thereby indicating the high burden of uncontrolled hypertension, and pointing to an immediate need to increase earlier detection of hypertension [9,10].

These baseline survey findings were pivotal in providing the key benchmarks to track NCD risk factors in the Ugandan population and informed the design of national-level NCD policy guidelines to address key NCD risk factors, including the importance of creating more public awareness about the NCDs and their risk factors [9,10]. Additional studies have described the barriers to hypertension prevention and management in rural settings, emphasized the need to increase the monitoring and screening of NCD risk factors at community levels through village health teams, and underscored broader access to essential NCD medicines, particularly in rural areas [11–13]. However, there have been a limited number of studies that examined the prevalence of hypertension and NCD risk factors together, along with factors associated with hypertension. In light of the suboptimal NCD risk factor surveillance efforts and recognizing the need for continuous monitoring of NCD risk factors, particularly undiagnosed hypertension, the study’s main objectives were to: (i) characterize the epidemiological profile of NCD risk factors; (ii) estimate the prevalence of hypertension; and (iii) identify factors associated with hypertension in a peri-urban and rural Ugandan population.

Methods

Study design, settings, and participants

This was a population-based cross-sectional survey of adults aged 18 years and older. It was nested in the longitudinal cohort of the Iganga-Mayuge Health and Demographic Surveillance System field site (IMHDSS) in eastern Uganda. Since 2004, IMHDSS staff conduct bi-annual visits to collect vital events and demographic information from a peri-urban and rural population of over 90,000 individuals residing in 17,000 households [14,15]. The site spans sixty-five villages across seven sub-counties, and it is served by sixteen health centers and two hospitals. Each village, household, and resident are enumerated by IMHDSS

staff, and these records are maintained in the IMHDSS database. Residents are assigned a unique ID that facilitates longitudinal monitoring. The database of all individuals, including their date of birth and sex, constituted the sampling frame for this study. Specifically, participants were eligible for the study if they were 18 years of age or older and had been IMHDSS residents for at least 6 months. Data collection took place between 06 November 2017 and 22 June 2018.

Ethics Statement

Ethical approval was obtained from the Makerere University School of Public Health Higher Degrees, Research and Ethics Committee, protocol (IRB: 00005876). They approved the study protocol and questionnaires. The Institutional Review Board of the Johns Hopkins Bloomberg School of Public Health exempted the study from ethical oversight and agreed to allow the study to rely on the Ethical Review Board of the Makerere University School of Public Health (IRB: 00000112 and 00000758).

Data collection

IMHDSS staff visited individuals selected from the IMHDSS database and obtained informed written consent. Consent and all data collection occurred at the participant’s residence. After consent was obtained, IMHDSS staff administered an abridged version of the World Health Organization’s (WHO) STEPwise approach to NCD risk factor surveillance (STEPS) instrument, using a tablet programmed with Open Data Kit (ODK). This instrument contained questions on demographics, tobacco use, alcohol consumption, dietary intake, and physical activity [16]. Following the administration of the questionnaire and at the same household visit, trained nurses measured the participant’s BP, weight, and height. We did not collect physical measurements from pregnant women.

BP was measured three times, spaced ten minutes apart, using the Omron 5 series upper arm monitor. For those who had physical measurements taken, if they were found to have high blood pressure but were in no acute danger of an event from BP (i.e., systolic BP of 140 mmHg to 179 mmHg, and a diastolic BP less than 120 mmHg), the research staff provided the participant with information on the importance of keeping optimal BP levels. However, if a participant was observed to be in hypertensive crisis (i.e., systolic or diastolic BP at least 180 or 120 mmHg, respectively), arrangements were made to transport the person to the nearest health facility if they were willing. According to Uganda’s Ministry of Health guidelines, government health facilities are expected to provide free health services, including medication for hypertension [17]. Anthropometric measurements of body height and weight were assessed using standard protocols, with participants standing upright, not wearing shoes, and wearing light-weight clothes. Height was measured twice, to the nearest 0.1 centimeter (cm). Body weight was measured twice to the nearest 0.1 kilogram (kg), using calibrated digital weighing scales. The participants were given feedback on the results of all the measurements immediately before the data collector left the household.

Outcomes

The average of the three BP readings was used to produce the final average systolic and diastolic BP estimates. Participants were classified as hypertensive and pre-hypertensive based on WHO-International Society of Hypertension (WHO-ISH) guidelines [18,19]. The primary outcome, hypertension, was defined as having a systolic or diastolic BP at least 140 or 90 mmHg, respectively, or if participants were currently on anti-hypertensive medication. Pre-hypertension was defined as having an systolic BP of 120 to 139 mmHg and/or diastolic BP of 80 to 89 mmHg [18,19].

Indicator variables for NCD risk factors were created in accordance with the STEPS analysis guide [16]. Current drinkers were defined as those who drank alcohol in the past 30 days. Heavy episodic drinking was defined as those who drank six or more standard alcoholic drinks on a single occasion in the past 30 days. Low fruit and vegetable consumption was defined as those who ate less than 5 servings of fruit and/or vegetables on average per day in a week. Given the low observed prevalence of tobacco smokers and smokeless tobacco use, we used current tobacco use (any type) in the regression models. The Global Physical Activity Questionnaire (GPAQ) analysis guide was used to create indicators for the level of physical activity [20]. Minutes spent doing physical activity were multiplied by a metabolic equivalent (MET) depending on the type of activity; 8 MET for vigorous intensity activities and 4 MET for moderate intensity activities. Insufficient physical activity, as defined by WHO, was defined as individuals who did not meet any of the following criteria: 150 minutes of moderate-intensity physical activity per week, 75 minutes of vigorous-intensity physical activity per week, or an equivalent combination of moderate- and vigorous-intensity physical activity accumulating at least 600 metabolic equivalent-minutes per week.

For anthropometric measurements, we used the average of the two measurements to produce the final standing height and body weight estimates. Body Mass Index (BMI; kilograms/meters^2 (kg/m^2)) was calculated and categorized into underweight (BMI <18.5), normal weight ($\geq$18.5 BMI < 25), overweight ($\geq$25 BMI < 30), and obese (BMI $\geq$30) in accordance with WHO recommendations [21].

Data analysis

First, we reported the sociodemographic characteristics of the respondents as per the status of hypertension (i.e., no hypertension, prehypertension, and hypertension); we used mean (with standard error (SE)) to report continuous variables and weighted percentages (%) with unweighted numbers (n) to report categorical variables. Continuous and categorical variables were compared by analysis of variance and chi-square tests, respectively. Then, we reported the overall and sex-stratified prevalence of risk factors for NCD as well as hypertension with 95% confidence intervals (CI). Lastly, to identify factors associated with hypertension, we calculated crude and adjusted prevalence ratios (PR) for each of the NCD risk factors using weighted Poisson regression with sample selection weights. Poisson regression was used due to convergence issues with log-binomial models. Only those demographic and NCD risk factor indicators that had significant associations in unadjusted analyses (p<0.5) were included in the adjusted (i.e., multivariable) model. From the list of tobacco-related variables, we only included those who were 'current tobacco users' as the unadjusted direction of association was similar for both smoked and smokeless tobacco use variables. Similarly, we only included one alcohol variable (i.e., current drinking) and one salt variable (i.e., add salt while eating). All statistical analyses were performed using Stata SE version 15. An alpha of 0.05 was assumed for all statistical tests of significance.

Sample size

Using the equation for sample size (n) with stratified random sampling, 385 IMHDSS adults were needed to estimate an indicator with an expected prevalence of 50% risk factor (p = 0.5), a 5 % margin-of-error (δ=0. 05), and a 5% type-I error (α=0. 05) for each of the 8 strata- male and female; age groups of 18-29 years, 30-44, 45-59, and 60+ years. n= $z_{1-\frac{\alpha}{2}}^2 \sigma^2/\delta^2$. Following eight age-sex strata, we needed a total of 3080 (i.e., 385 * 8) participants.

Results

Table 1: Comparison of the study sample according to hypertension status

Variable	Overall	Blood pressure levels			p-value
		Normal	Prehypertension	Hypertension	

Age (in year)	Mean (SE)	35.3 (0.1)	31.7 (0.3)	33.8 (0.3)	48.7 (0.7)	<0.001
	18-29	46.2 (694)	57.7 (294)	49.4 (362)	14 (38)	<0.001
	30-44	29 (727)	26.9 (220)	30 (371)	30.7 (136)	
	45-59	16.3 (823)	12 (203)	14.8 (359)	29.3 (261)	
	60+	8.5 (883)	3.5 (121)	5.8 (296)	26 (466)	
Sex	Female	49.4 (1626)	58.8 (485)	42.3 (639)	50.8 (502)	<0.001
	Male	50.6 (1501)	41.2 (353)	57.7 (749)	49.2 (399)	
Edu-cation	No education	9.1 (577)	6.7 (112)	7.8 (220)	17.5 (245)	<0.001
	Primary	43.6 (1480)	41.6 (387)	43.3 (655)	48.5 (438)	
	Secondary	38.5 (850)	42 (275)	40.7 (416)	25.4 (159)	
	Above secondary	8.8 (220)	9.7 (64)	8.2 (97)	8.6 (59)	
Marital status	Never married	28.5 (435)	32.4 (166)	32.4 (240)	9.9 (29)	<0.001
	Married	60.5 (2020)	59.9 (550)	58.6 (901)	67.4 (569)	
	Divorced/separated	5.6 (241)	4.8 (54)	5.3 (104)	8.1 (83)	
	Widowed	5.3 (431)	2.9 (68)	3.8 (143)	14.6 (220)	
Location	Rural	58.7 (2035)	63.9 (581)	54.6 (839)	60.3 (615)	<0.001
	Peri-urban	41.3 (1092)	36.1 (257)	45.4 (549)	39.7 (286)	
Wealth index	Lowest	19.9 (632)	21.4 (184)	17.4 (229)	23.8 (219)	0.21
	Lower	20.7 (520)	19.3 (133)	22.5 (251)	18.6 (136)	
	Middle	21.6 (576)	21.7 (158)	21.9 (262)	20.8 (156)	
	Higher	22.5 (506)	22 (134)	23.6 (236)	20.3 (136)	
	Highest	15.3 (321)	15.7 (85)	14.6 (141)	16.5 (95)	

Table 1 shows the sociodemographic characteristics of the respondents as per their hypertension status. A total of 3220 people were included in the analyses; the mean age of the participants was 35.3 years (SE: 0.1); the mean age (SE) of hypertensive people was higher than those with normal BP or prehypertension, 48.7 (0.7), 33.8 (0.3), and 31.7 (0.3), respectively (p<0.001). About 49.4% of the people were female. The proportion of people without any formal education was 9.1%; this proportion was higher among those with hypertension (17.5%) than people without hypertension (6.7%) or prehypertension (7.8%). A majority of the respondents were married (60.5%) or were living in rural regions (58.7%); these characteristics also differed by hypertension status. The wealth index did not differ by hypertension status. We also reported the demographics by sex (S1 Table).

Table 2. Weighted estimates of noncommunicable disease risk factors by sex

Indicator	Female	Male	Both sexes
Tobacco use			
Current tobacco smoker	0.7 (0.4,1.1)	7.6 (6.3,9.1)	4.0 (3.4,4.8)
Daily tobacco smoker	0.3 (0.2,0.6)	5.0 (3.9,6.3)	2.6 (2.1,3.2)
Current smokeless tobacco	0.1 (0.1,0.3)	1.4 (0.8,2.4)	0.7 (0.5,1.2)
Daily smokeless tobacco	0.1 (0.0,0.2)	0.6 (0.3,1.4)	0.3 (0.2,0.7)
Current tobacco use (any)	0.7 (0.4,1.1)	8.4 (7.0,10.0)	4.4 (3.7,5.3)
Alcohol consumption			
Current drinker (past 30 days)	4.6 (3.6,5.8)	11.1 (9.4,13.0)	7.7 (6.7,8.8)

Heavy episodic drinking*	4.0 (3.0,5.2)	9.4 (7.8,11.2)	6.6 (5.7,7.7)
Diet			
Low fruit & vegetable consumption†	98.1 (97.1,98.7)	98.9 (98.1,99.3)	98.5 (97.9,98.9)
Always/often add salt while eating	4.1 (3.1,5.6)	5.0 (3.9,6.6)	4.6 (3.7,5.6)
Always/often add salt while cooking	45.5 (42.6,48.5)	48.1 (45.0,51.1)	46.8 (44.7,48.9)
Always/often eat processed foods high in salt	7.1 (5.7,8.9)	13.4 (11.4,15.8)	10.2 (8.9,11.6)
Physical Activity			
Insufficient physical activity‡	9.9 (8.4,11.7)	5.9 (4.7,7.3)	8.0 (7.0,9.1)
Body Mass Index (BMI; kg/m^2) §			
Underweight (BMI< 18.5)	2.0 (1.4,2.8)	2.3 (1.6,3.3)	2.1 (1.6,2.7)
Normal (≥18.5 BMI < 25)	52.6 (49.5,55.6)	70.5 (67.8,73.2)	61.7 (59.6,63.7)
Overweight (≥25 BMI < 30)	30.7 (28.0,33.6)	23.1 (20.7,25.7)	26.9 (25.0,28.8)
Obese (BMI ≥30)	14.7 (12.8,16.8)	4.1 (3.1,5.2)	9.3 (8.2,10.5)

Data are prevalence (95%CI) and are weighted to 2016 IM-HDSS population by age-sex. * Percent of population who had six or more alcoholic drinks in one sitting in the past month. †Defined as those who ate less than 5 servings of fruit and/or vegetables on average per day. ‡Defined as not achieving 150 minutes of moderate-intensity physical activity OR 75 minutes of vigorous-intensity physical activity OR an equivalent combination of moderate- and vigorous-intensity physical activity achieving at least 600 MET-minutes. § Excludes pregnant women. Abbreviations: kg, kilogram; m, meter.

Table 2 presents the population prevalence of tobacco use, alcohol consumption, diet, physical activity, and BMI weighted to 2016 IMHDSS population by age and sex. Males had a higher prevalence of current tobacco smokers (7.6%, 95 %CI: 6.3%-9.1%), current smokeless tobacco users (1.4%, 95% CI: 0.81%-2.4%), current drinkers (11.1%, 95% CI: 9.4%-13.0%), and heavy episodic drinking (9.4%, 95% CI: 7.8%-11.2%), than females, (0.65%, 95% CI: 0.39%-1.1%), (0.13%, 95% CI: 0.06%-0.31%), (4.6%, 95% CI: 3.6%-5.8%), and (4.0%, 95% CI: 3.1%-5.2%) respectively. Females had higher prevalence of insufficient physical activity (9.9%, 95% CI: 8.4%-11.7%), and obesity (14.7%, 95% CI: 12.9%-16.8%), as compared to males, (5.9%, 95% CI: 4.7%-7.2%) and (4.1%, 95% CI: 3.1%-5.2%), respectively. Population prevalence estimates for low fruit and vegetable consumption, adding salt while eating, and adding salt while cooking were similar between males and females; the overall prevalence for these indicators, respectively, was (98.4%, 95% CI: 97.9%-98.9%), (4.6%, 95% CI: 3.7%-5.6%), and (46.8%, 95% CI: 44.7%-48.8%).

Table 3. Weighted distribution of blood pressure levels by sex

Indicator	Female	Male	Both sexes
**Blood pressure field reading**			
Normal blood pressure*	40.5 (37.6-43.5)	27.7 (25.0-30.6)	34.0 (32.0-36.1)
Pre-hypertension†	41.9 (38.9-44.9)	55.7 (52.7-58.6)	48.8 (46.8-51.0)
Hypertension‡	17.6 (15.9-19.4)	16.6 (14.8-18.6)	17.1 (15.9-18.4)
**Of those who had hypertension**	n=502	n=399	n=901
Newly identified hypertension case§	52.4 (47.9-56.8)	74.9 (70.4-79.1)	62.4 (59.1-65.5)
Uncontrolled hypertension¶	96.4 (93.8-98.0)	98.4 (96.2-99.3)	97.4 (95.8-98.4)
Controlled hypertension**	3.6 (2.0-6.2)	1.6 (0.69-3.8)	2.6 (1.6-4.2)

Data are prevalence (95%CI) and are weighted to 2016 IM-HDSS population by age-sex. Excludes pregnant women. * SBP <120 mm Hg and DBP <80 mm Hg and not taking antihypertensive medication. † Either SBP between 120 and 139 mmHg or DBP between 80 and 89 mmHg. ‡SBP ≥ 140 and/or DBP ≥ 90 mmHg or currently on medication for raised blood pressure. § SBP ≥ 140 and/or DBP ≥ 90 mmHg and not currently on medication for raised blood pressure and never been diagnosed with high blood pressure

¶SBP ≥ 140 and/or DBP ≥ 90 mmHg and not currently on medication for raised blood pressure. ** SBP< 140 and DBP <90 mmHg and currently on medication for raised blood pressure.

Overall, the weighted prevalence of hypertension was estimated to be 17.1% (95% CI: 15.9%-18.4%), and was similar between males (16.6%, 95% CI: 14.8%-18.6%) and females (17.6%, 95% CI: 15.9%-19.4%; **Table 3**). A higher prevalence of pre-hypertension, defined as having either systolic BP between 120 and 139 mmHg or diastolic BP between 80 and 89 mmHg was observed in males (55.7% 95% CI: 52.7%-58.7%) as compared to females (41.9% 95% CI: 38.9%-44.9%). Of the 399 male and 502 female hypertensive participants, 83.3% and 57.8% were newly identified as being hypertensive (i.e., had not received a prior hypertension diagnosis). The population prevalence of uncontrolled hypertension, i.e., being hypertensive but not on medications was high in both males (98.4% 95%CI: 96.2%-99.3%) and females (96.4% 95%CI: 93.8%-98.0%).

Table 4. Crude and adjusted prevalence ratios for hypertension

Variable		Unadjusted		Adjusted	
		CPR	p-value	APR	p-value
Age (year, ref.: 18-29 years)	30-44	3.5*** (2.4,4.9)	<.001	3.1*** (2.2,4.4)	<.001
	45-59	5.9*** (4.2,8.2)	<.001	5.2*** (3.7,7.3)	<.001
	60+	10.1*** (7.3,13.9)	<.001	8.9*** (6.4,12.5)	<.001
Male gender (ref.: Female)		0.9 (0.8,1.1)	.44		
Education (ref.: No education)	Primary	0.6*** (0.5,0.7)	<.001	1.0 (0.9,1.2)	.89
	Secondary	0.3*** (0.3,0.4)	<.001	1.0 (0.8,1.2)	.72
	Above Secondary	0.5*** (0.4,0.7)	<.001	1.0 (0.7,1.4)	.95
Location, Peri-urban (ref.: Rural)		0.9 (0.8,1.1)	.45		
Current tobacco smoker (ref = No)		2.0*** (1.5,2.6)	<.001		
Daily tobacco smoker (ref = No)		1.6* (1.1,2.3)	.024		
Current smokeless tobacco user (ref = No)		2.1* (1.1,4.0)	.025		
Daily smokeless tobacco user (ref = No)		0.3 (0.1,1.4)	.13		
Current tobacco (any) user (ref = No)		1.9*** (1.5,2.5)	<.001	1.2 (0.9,1.6)	.16
Daily tobacco (any) user (ref = No)		1.4 (1.0,2.1)	.064		
Drank alcohol in past month (ref = No)		2.1*** (1.7,2.5)	<.001	1.6*** (1.3,2.0)	<.001
Heavy episodic drinking* (ref = No)		2.0*** (1.6,2.5)	<.001		
Low fruit & vegetable consumption (ref = No)		1.1 (0.6,1.9)	.86		.
Add salt while eating (ref: Never/rarely)	Sometimes	0.8* (0.7,1.0)	.024	1.0 (0.9,1.2)	.68
	Often/always	1.3 (0.9,1.8)	.2	1.6* (1.1,2.2)	.016
Add salt while cooking	Sometimes	0.7*** (0.6,0.9)	<.001		
	Often/always	0.7** (0.6,0.9)	.002		
Eat processed foods high in salt	Sometimes	0.8** (0.7,0.9)	.004		
	Often/always	0.7 (0.5,1.0)	.068		
Poor physical activity (ref = No)		1.8*** (1.5,2.2)	<.001	1.3** (1.1,1.6)	.008
Body mass index (ref: <25.0 kg/m2)	25.0-29.9	1.4*** (1.2,1.7)	<.001	1.3** (1.1,1.5)	.006
	30 or more	2.7*** (2.2,3.3)	<.001	2.0*** (1.6,2.4)	<.001

Abbreviations: CPR, crude prevalence ratio; APR, adjusted prevalence ratio.

Table 4 presents a summary of the unadjusted and adjusted associations of potential risk factors for hypertension. Several factors were determined to be significantly associated with hypertension in adjusted models, including age, drinking alcohol in the last month, adding salt while eating, poor physical activity, and being overweight or obese. For instance, compared to 18-29-year-old people, those with 30-44 (adjusted PR (APR): 3.1, 95% CI: 2.2-4.4, p<0.001), 45-59 (APR: 5.2, 95% CI: 3.7-7.3, p<0.001), and 60+ years of age (APR: 8.9, 95% CI: 6.4-12.5, p<0.001) had a higher prevalence of hypertension. Those with current alcohol drinking (APR: 1.6, 95% CI: 1.3-2.0, p<0.001) or poor physical activity (APR: 1.3, 95% CI: 1.1-1.6, p<0.001) also had a higher prevalence of hypertension compared to people without alcohol drinking or sufficient physical activity. S2 and S3 Tables show the risk factors by sex.

Discussion

Findings from this household survey reveal a high prevalence of NCD risk factors in a peri-urban and rural population in Eastern Uganda. We found that about two-thirds of the respondents had higher than normal BP (i.e., either prehypertension or hypertension). Most respondents were not taking an adequate amount of fruits/vegetables and over one-third were either overweight or obese. We also identified several risk factors for hypertension, including older age, alcohol drinking, low physical activity, overweight, and obesity. This study adds to the growing body of literature investigating the burden of hypertension and NCD risk factors in LMICs.

The low fruit and vegetable consumption reported by our study was similar to the 2014 Ugandan STEPS NCD survey. The survey reported that nearly 88% of the participants had low fruit and/or vegetable consumption (defined as <5 servings on average per day) [10]. **S4 and S5 Tables** present a side-by-side comparison of 2014 national survey estimates with IMHDSS estimates for NCD risk factors in males and females, respectively, by age. Similarly, a recent cross-sectional national survey in Uganda similarly found that among participants from the Eastern region, the same region as Iganga-Mayuge, only 7.5% consumed ≥ 5 servings of fruits or vegetables per day [22]. Our study reveals a lower consumption of fruit and vegetables, which may be attributed to the dietary changes associated with increasing urbanization of rural and peri-urban areas, and perhaps a regional focus on producing commercial agricultural produce – for examples, cereals and roots – as opposed to fruits and vegetables [23]. A diet low on fruits and vegetables, but high on energy-rich foods are expected to be associated with higher levels of adiposity, body weight, and blood pressure [24,25].

Another important finding was the high prevalence of hypertension within the Iganga Mayuge community, with nearly 17% and 50% of those participants having hypertension and pre-hypertension, respectively. Though the prevalence of hypertension was lower than the 2014 Ugandan STEPS NCD, which reported that 26.5% of adults aged 18 to 64 years had it [10], in a peri-urban environment of Iganga-Mayuge the onset of hypertension, a cardiometabolic risk factor may observed over longer time periods following increases in body fat [25]. The high percentage of pre-hypertension in this community is worth noting, especially as some studies estimate that nearly 1 in 3 people with pre-hypertension develop hypertension within 4 years [26]. In our study, only 9.4% of survey participants had ever been diagnosed with hypertension, and nearly 60% had never had their BP measured. Low levels of awareness about adults' own hypertension status have been documented in Uganda [9,24], including in the 2014 Ugandan STEPS survey which reported that only about 8% of participants were aware of their hypertension status [10], and in other Sub-Saharan countries [27,28].

The low levels of awareness of one's hypertension status may reflect the health system's limited capacity to provide timely diagnostic, preventive, and treatment services to the general population [29]. Even within

our study, we frequently had to refer participants who had been diagnosed with hypertension to the nearest district hospital because primary and community health centers often lacked diagnostic devices or were experiencing stockouts for hypertension treatment. To improve the availability of services in health facilities, the Ugandan Ministry of Health and NCD partners are already taking steps to improve population-level awareness around NCDs by updating clinical guidelines for managing major NCD conditions as well as developing and implementing programs to increase population-level awareness about NCDs and their risk factors [30]. While these activities demonstrate progress, deep-seated concerns remain among both government and external actors around the government's commitment to increase funding for NCD prevention and control in Uganda [7,30].

Our study has several strengths. To our knowledge, this is the first study in Uganda since the 2014 Ugandan STEPS survey that systematically measured the major NCD risk factors in any region. In addition, a large population-based sample allows us to examine risk factors for NCDs in a peri-urban region in Uganda. Thirdly, our team of well-trained and experienced data collectors was critical in minimizing errors associated with taking anthropometry and BP measurements. Finally, our study aimed to reduce non-response rates by visiting households at least three times and at varying time points to reach the target survey participant.

Despite these advantages, our study is not without limitations. Though we can examine associations among different factors affecting NCDs, the cross-sectional design restricts us from making any causal inferences. Secondly, there may be potential for recall bias as many risk factors are self-reported by the participant. We further recognize that participants may have had difficulty in both assessing and recalling responses to certain questions, for example, around the actual number of servings of fruits and vegetables they consume on average in a day. A validation study may have addressed this concern; however, we tried to minimize this effect by using a survey that was based on a validated questionnaire. Thirdly, we recognize the potential misclassification of BP; however, we safeguarded against this by taking these BP measurements.

Conclusion

Our study found a high prevalence of NCD risk factors in a peri-urban and rural population in Eastern Uganda, including indicators related to tobacco, diet, alcohol, and physical activity. We observed a high prevalence of pre-hypertension/hypertension highlighting the need to implement and scale-up population-level strategies to increase awareness about leading NCD risk factors, especially the indicators studied here. A concomitant increase in health system's capacity to increase routine screening for hypertension and the provision of timely treatment will be critical for managing NCDs.

Acknowledgement

We would like to thank all data collectors, administrators and staff at Iganga-Mayuge Health and Demographic Surveillance Site for their support with the planning and implementation of the data collection activities.

Author Contributions

Conceptualization: Dustin Gibson
Data curation: Tryphena Nareeba and Collins Gyehazo
Formal analysis: Dustin Gibson and Gulam Muhammed Al Kibria
Funding acquisition: Dustin Gibson and Adnan A Hyder
Investigation: Edward Galiwango and Judith Kaija Nanyonga
Methodology: Dustin Gibson, Dan Kajungu, and Gulam Muhammed Al Kibria,
Project administration: Dan Kajungu
Resources: Tryphena Nareeba and Edward Galiwango

Software: Dustin Gibson,
Supervision: Elizeus Rutebemberwa, Dan Kajungu, Dustin Gibson
Writing – original draft: Dustin Gibson and Anikita Meghani
Writing – review & editing: Gulam Muhammed Al Kibria, Charles Ssemagabo, Elizeus Rutebemberwa, Adaeze Wosu, Adnan A Hyder, George W Pariyo, and Dan Kajungu

Funding
Research reported in this publication was supported by the Fogarty International Center of the National Institutes of Health under Award Number (DUNS Number001910777, Project # 1R21TW010415-01). The content is solely the responsibility of the authors and does not necessarily represent the official views of the Fogarty International Center nor the National Institutes of Health.

References

1. Roth GA, Abate D, Abate KH, Abay SM, Abbafati C, Abbasi N, et al. Global, regional, and national age-sex-specific mortality for 282 causes of death in 195 countries and territories, 1980–2017: a systematic analysis for the Global Burden of Disease Study 2017. The Lancet. 2018;392: 1736–1788. doi:10.1016/S0140-6736(18)32203-7

2. Wang H, Naghavi M, Allen C, Barber RM, Bhutta ZA, Carter A, et al. Global, regional, and national life expectancy, all-cause mortality, and cause-specific mortality for 249 causes of death, 1980–2015: a systematic analysis for the Global Burden of Disease Study 2015. The Lancet. 2016;388: 1459–1544. doi:10.1016/S0140-6736(16)31012-1

3. Forouzanfar MH, Liu P, Roth GA, Ng M, Biryukov S, Marczak L, et al. Global Burden of Hypertension and Systolic Blood Pressure of at Least 110 to 115 mm Hg, 1990-2015. JAMA. 2017;317: 165–182. doi:10.1001/jama.2016.19043

4. Forouzanfar MH, Afshin A, Alexander LT, Anderson HR, Bhutta ZA, Biryukov S, et al. Global, regional, and national comparative risk assessment of 79 behavioural, environmental and occupational, and metabolic risks or clusters of risks, 1990–2015: a systematic analysis for the Global Burden of Disease Study 2015. The Lancet. 2016;388: 1659–1724. doi:10.1016/S0140-6736(16)31679-8

5. Uganda Bureau of Statistics, ICF International. Uganda Demographic and Health Survey 2016. Available: https://dhsprogram.com/pubs/pdf/FR333/FR333.pdf

6. Essue BM, Kapiriri L. The unfunded priorities: an evaluation of priority setting for noncommunicable disease control in Uganda. Glob Health. 2018;14: 22. doi:10.1186/s12992-018-0324-2

7. Schwartz JI, Guwatudde D, Nugent R, Kiiza CM. Looking at non-communicable diseases in Uganda through a local lens: an analysis using locally derived data. Glob Health. 2014;10: 77. doi:10.1186/s12992-014-0077-5

8. Institute for Health Metrics and Evaluation (IHME). GBD Compare Data Visualization. Seattle, WA: University of Washington; 2020. Available: http://vizhub.healthdata.org/gbd-compare

9. Guwatudde D, Mutungi G, Wesonga R, Kajjura R, Kasule H, Muwonge J, et al. The Epidemiology of Hypertension in Uganda: Findings from the National Non-Communicable Diseases Risk Factor Survey. Kokubo Y, editor. PLOS ONE. 2015;10: e0138991. doi:10.1371/journal.pone.0138991

10. Wu Y, Cowan M. Uganda: STEPs 2014. Geneva, Switzerland; 2014. Available: https://extranet.who.int/ncdsmicrodata/index.php/catalog/633

11. Nanono J, Neupane D, Ssekamatte T, Ahumuza E, Kasujja FX, Rutebemberwa E. Service Availability and Readiness of Primary Care Health Facilities Offering Hypertension Diagnosis Services in Wakiso District, Uganda, 2019. Prev Chronic Dis. 2023;20: 220236. doi:10.5888/pcd20.220236

12. Majumdar U, Nanyonga Clarke R, Moran AE, Doupe P, Gadikota-Klumpers DD, Gidio A, et al. Hypertension screening, prevalence, treatment, and control at a large private hospital in Kampala, Uganda: A retrospective analysis. Amaral AFS, editor. PLOS Glob Public Health. 2022;2: e0000386. doi:10.1371/journal.pgph.0000386

13. Chang H, Hawley NL, Kalyesubula R, Siddharthan T, Checkley W, Knauf F, et al. Challenges to hypertension and diabetes management in rural Uganda: a qualitative study with patients, village health team members, and health care professionals. Int J Equity Health. 2019;18: 38. doi:10.1186/s12939-019-0934-1

14. Kajungu D, Hirose A, Rutebemberwa E, Pariyo GW, Peterson S, Guwatudde D, et al. Cohort Profile: The Iganga-Mayuge Health and Demographic Surveillance Site, Uganda (IMHDSS, Uganda). Int J Epidemiol. 2020;49: 1082–1082g. doi:10.1093/ije/dyaa064

15. Makerere University Center for Health and Population Research. Demographic Surveillance. 2019. Available: https://muchap.org/what-we-do/demographic-surveillance

16. World Health Organization. STEPwise Approach to Surveillance (STEPS). 2022. Available: https://www.who.int/teams/noncommunicable-diseases/surveillance/systems-tools/steps

17. The Republic of Uganda- Ministry of Health. Uganda Clinical Guidelines 2016 - National Guidelines for Management of Common Conditions. 2016. Available: https://www.prb.org/wp-content/uploads/2018/05/Uganda-Clinical-Guidelines-2016-National-Guidelines-for-Management-of-Common-Conditions.pdf

18. Chalmers J, MacMahon S, Mancia G, Whitworth J, Beilin L, Hansson L, et al. 1999 World Health Organization-International Society of Hypertension Guidelines for the management of hypertension. Guidelines sub-committee of the World Health Organization. Clin Exp Hypertens. 1999;21: 1009–1060. doi:10.3109/10641969909061028

19. Chobanian AV, Bakris GL, Black HR, Cushman WC, Green LA, Izzo JL, et al. The Seventh Report of the Joint National Committee on Prevention, Detection, Evaluation, and Treatment of High Blood Pressure: the JNC 7 report. JAMA. 2003;289: 2560–2572. doi:10.1001/jama.289.19.2560

20. World Health Organization. Global Physical Activity Questionnaire (GPAQ) Analysis Guide. Geneva, Switzerland; Available: https://www.who.int/docs/default-source/ncds/ncd-surveillance/gpaq-analysis-guide.pdf

21. World Health Organization. Body Mass Index Classification. 2020. Available: http://apps.who.int/bmi/index.jsp?introPage=intro_3.html

22. Kabwama SN, Bahendeka SK, Wesonga R, Mutungi G, Guwatudde D. Low consumption of fruits and vegetables among adults in Uganda: findings from a countrywide cross-sectional survey. Arch Public Health. 2019;77: 4. doi:10.1186/s13690-019-0332-6

23. Riha J, Karabarinde A, Ssenyomo G, Allender S, Asiki G, Kamali A, et al. Urbanicity and Lifestyle Risk Factors for Cardiometabolic Diseases in Rural Uganda: A Cross-Sectional Study. Razak F, editor. PLoS Med. 2014;11: e1001683. doi:10.1371/journal.pmed.1001683

24. Musinguzi E, Kikafunda J, Kiremire B. Utilization of indigenous food plants in Uganda: A case study of south-western Uganda. Afr J Food Agric Nutr Dev. 2011;6. doi:10.4314/ajfand.v6i2.71738

25. Kinra S, Andersen E, Ben-Shlomo Y, Bowen L, Lyngdoh T, Prabhakaran D, et al. Association Between Urban Life-Years and Cardiometabolic Risk: The Indian Migration Study. Am J Epidemiol. 2011;174: 154–164. doi:10.1093/aje/kwr053

26. Vasan RS, Larson MG, Leip EP, Kannel WB, Levy D. Assessment of frequency of progression to hypertension in non-hypertensive participants in the Framingham Heart Study: a cohort study. The Lancet. 2001;358: 1682–1686. doi:10.1016/S0140-6736(01)06710-1

27. Ataklte F, Erqou S, Kaptoge S, Taye B, Echouffo-Tcheugui JB, Kengne AP. Burden of Undiagnosed Hypertension in Sub-Saharan Africa: A Systematic Review and Meta-Analysis. Hypertension. 2015;65: 291–298. doi:10.1161/HYPERTENSIONAHA.114.04394

28. Hendriks ME, Wit FWNM, Roos MTL, Brewster LM, Akande TM, De Beer IH, et al. Hypertension in Sub-Saharan Africa: Cross-Sectional Surveys in Four Rural and Urban Communities. Atashili J, editor. PLoS ONE. 2012;7: e32638. doi:10.1371/journal.pone.0032638

29. Katende D, Mutungi G, Baisley K, Biraro S, Ikoona E, Peck R, et al. Readiness of Ugandan health services for the management of outpatients with chronic diseases. Trop Med Int Health. 2015;20: 1385–1395. doi:10.1111/tmi.12560

30. Meghani A, Ssemugabo C, Pariyo G, Hyder AA, Rutebemberwa E, Gibson DG. Curbing the Rise of Noncommunicable Diseases in Uganda: Perspectives of Policy Actors. Glob Health Sci Pract. 2021;9: 149–159. doi:10.9745/GHSP-D-20-00051

Click here to access/download
Supporting Information
Supp_Tables_Hypertension_NCD_Ugx.docx

Click here to access/download
Supporting Information
MoP_NCD study_IRB approval letter.pdf

Click here to access/download
Supporting Information
IRB reliance form_signed and executed.pdf
