# Supplementary material for: The epidemiology of behavioral risk factors for noncommunicable disease and hypertension: A cross-sectional study from Eastern Uganda
Source: PLOS Glob Public Health. 2024 Jun 17;4(6):e0002998. doi: 10.1371/journal.pgph.0002998 (PMC11182527; doi:10.1371/journal.pgph.0002998)
Supplement: Attachment — Submitted filename: Response Letter.docx [file pgph.0002998.s009.docx]

Dear Editor,

Thank you for providing us the opportunity to upload a revised version of the manuscript. We want to thank the reviewers for their valuable time. Here I have added itemized responses to comments/questions. I believe we were able to address all your concerns. Please see the responses below

**Reviewer #1:**

**Comment 1:** The manuscript seems to be technically sound, and the data supports the conclusions. The methods used can be clearly understood and the study can be replicated. Appropriate ethical considerations have been taken care off. Conclusions are appropriate and drawn based on the data presented. Refer to the attached manuscript for recommendations.

In Title: Disease, Behavioral

***Response:*** *We have edited the title as the reviewer suggested. Thanks for the valuable suggestion and for the kind words.*

**Comment 2:** Check the diastolic BP values stated.

***Response:*** *We have double-checked this and found this correct, thanks so much again!*

**Reviewer #2:**

**Comment 1:** Abstract: Consider revising your abstract to be reader friendly by indicating and maybe reducing font size of statistical indicators and figures .
***Response:*** *Thank you for the comment. We agree with the reviewer and edited the abstract*.

**Comment 2:** Introduction: In the problem statement , authors raised the issue of suboptimal NCD risk factors surveillance efforts and in their methods said the study was nested in a longitudinal cohort( surveillance ) data . So which gap is the paper addressing since surveillance data was available?

***Response****: Thank you for the comment. We aimed to asses the risk factors as highlighted in the last sentence. We have added the following sentence: “*Therefore, studies required to examine the risk factors in countries with increasing NCD burden, including Uganda.*”*

Reviewer #3: A very good study and timely,
**Comment 1:** A few comments to note, the authors have indicated that the BP was measured three times but did not indicate which arm was used to measure the BP, Art is known that BP differs in the two arms. How can the reader confirm that BP was measured consistently in the same arm for all the respondents?
***Response:*** *Yes, it was measured in the left arm, we have added that now: “*The upper arm of the left side was used.*” Thanks so much for the comment!*

**Comment 2:** Why were pregnant women excluded yet they have more challenges with BP? And why were they part of the sample if BP and weight were not going to be measured?
***Response:*** *Pregnant mothers were excluded as their increase BP could be attributed to pregnancy instead of having a real hypertension.*

**Comment 3:** it will be good to capture in the literature the pros and cons of home BP measurement vs health facility.

***Response:*** *We have added that as a limitation. Thank you for the suggestion.*

**Comment 4:** It will be good to understand how a serving was defined to allow the respondent to recall close to accurately. How was serving of vegetables designed given the challenges of dietary recalls.

***Response:*** *Thanks so much!* *We have added the explanation:* “A standard serving of fruit can be a medium-sized apple, banana, orange, or guava, or around half a cup of cooked or chopped fruit. Similarly, a typical serving of vegetables might be about a cup of leafy greens or salad, or half a cup of cooked or chopped vegetables.”

Dear Editor, we believe we were able to address all the comments. Thank you for your consideration!

Kind regards,

Gulam Muhammed Al Kibria, MBBS, MSPH, PhD

Johns Hopkins University
